# Nanomaterial-based microelectrode arrays for in vitro bidirectional brain–computer interfaces: a review

**DOI:** 10.1038/s41378-022-00479-8

**Published:** 2023-01-30

**Authors:** Yaoyao Liu, Shihong Xu, Yan Yang, Kui Zhang, Enhui He, Wei Liang, Jinping Luo, Yirong Wu, Xinxia Cai

**Affiliations:** 1 State Key Laboratory of Transducer Technology, Aerospace Information Research Institute, Chinese Academy of Sciences, Beijing, 100190 China; 2 School of Electronic, Electrical and Communication Engineering, University of Chinese Academy of Sciences, Beijing, 100049 PR China

**Keywords:** Biosensors, Biosensors

## Abstract

A bidirectional in vitro brain–computer interface (BCI) directly connects isolated brain cells with the surrounding environment, reads neural signals and inputs modulatory instructions. As a noninvasive BCI, it has clear advantages in understanding and exploiting advanced brain function due to the simplified structure and high controllability of ex vivo neural networks. However, the core of ex vivo BCIs, microelectrode arrays (MEAs), urgently need improvements in the strength of signal detection, precision of neural modulation and biocompatibility. Notably, nanomaterial-based MEAs cater to all the requirements by converging the multilevel neural signals and simultaneously applying stimuli at an excellent spatiotemporal resolution, as well as supporting long-term cultivation of neurons. This is enabled by the advantageous electrochemical characteristics of nanomaterials, such as their active atomic reactivity and outstanding charge conduction efficiency, improving the performance of MEAs. Here, we review the fabrication of nanomaterial-based MEAs applied to bidirectional in vitro BCIs from an interdisciplinary perspective. We also consider the decoding and coding of neural activity through the interface and highlight the various usages of MEAs coupled with the dissociated neural cultures to benefit future developments of BCIs.

## Graphical abstract

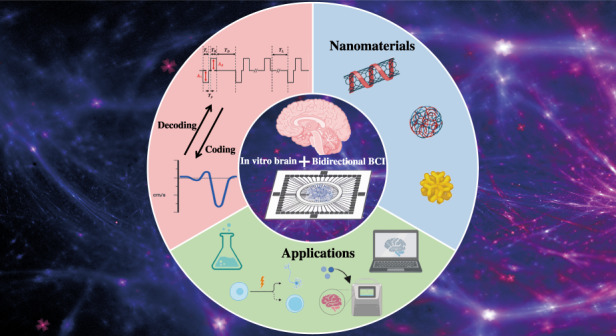

## Introduction

Based on the actuations of billions of neurons, the brain ensures individual survival in the rapidly changing environment and is responsible for behavior, cognition and emotion^1^. A brain–computer interface (BCI) builds a direct bridge between the brain and the surroundings to understand, recover and boost neurologic functions, which is also promising for utilizing human intelligence, essentially a subtle computing power^2^. BCIs have been in the limelight for years, and the emerging “metaverse”, where everyone is immersed in virtual reality, is another primary usage scenario of the technology^3–5^. In particular, an in vitro bidirectional BCI, which can both monitor and modulate a brain on a chip, is distinguished by its variety of potential applications^6–8^. Such devices help overcome the obstacles of in vivo neuroprostheses by decreasing complications and resulting in the mutual benefit of both technologies^9^. Additionally, the ex vivo BCI also reveals important biological mechanisms and widens the applications of micro biosensors, contributing to biological computers and neuromorphological computing^10^. This review aims to facilitate the fabrication of in vitro bidirectional BCIs and popularize the technology by explaining the underlying mechanism of bidirectional communication.

A microelectrode array (MEA), a tool for detecting cellular electrical activities, is the key to the ex vivo biosensing system. It is composed of ten to several thousand electrodes arranged on a rigid or flexible base, forming a customized pattern, which is also known as multielectrode array. The diameters of the published microelectrodes range from 5 to 50 *μm*, roughly corresponding to the scale of neurites or neuron somas. Thus, the changes in the ion concentration of the microenvironment arising from individual neuronal activity can be registered and sorted as spikes or local field potentials (LFP)^11^. MEAs achieve multisite readouts of neuronal ensembles noninvasively and at cellular resolution, which is low-cost and label-free. And Thomas et al.^12^ demonstrated the first in vitro test using the device on brain tissue in 1972 even before Vidal et al.^13^ proposed the innovative BCI in 1973. In summary, MEAs are superior for in vitro brain research.

Methods for improving MEA performance abound, a ubiquitous and effective one of which is the introduction of nanomaterials^14^. Bioelectrical interfaces with nanostructures generally possess improved mechanical compliance and biocompatibility^15^. Additionally, the nanocoating on electrodes increases the electrochemical reaction area and therefore improves the probing and stimulating performances^16^.

**Fig. 1:**
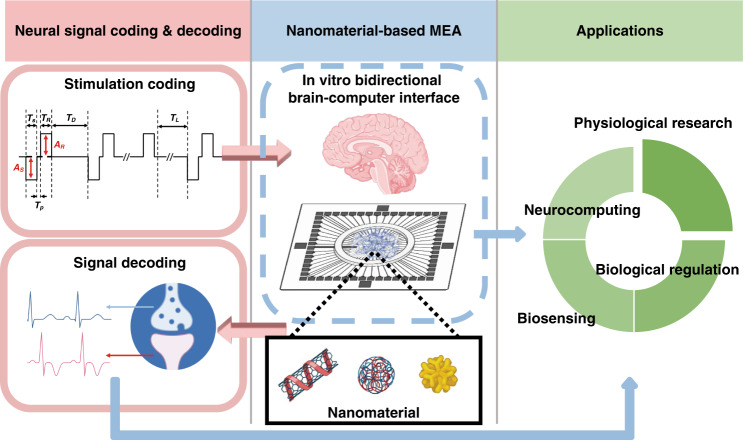
Nanomaterial-based MEA for an in vitro bidirectional BCI. Bidirectional communications between neuronal culture and the outside world are achieved by nanomaterial-based MEAs through stimulation coding and signal decoding, which can be further applied to physiological research, biological regulation, biosensing and neurocomputing (created with http://BioRender.com)

This review is generally divided into four sections, covering device manufacturing, data analysis, application prospects and development direction of the in vitro bidirectional BCI (Fig. 1). To appeal to researchers from an interdisciplinary background, the current progress of in vitro BCIs and the basic biological background related to in vitro cultured neural networks are discussed in the introduction section. First, the fabrication of MEAs is addressed with the evaluation criteria to assess the devices. Special emphasis is placed on nanomaterial-based modifications of the MEA for bidirectional communication. The second section discusses the analysis methods of the data from MEAs at the cellular level, population level and network level, together with paradigms of injecting information into isolated neural cultures. These topics lay the foundation for the rich applications of bidirectional in vitro BCIs in the third part. The last part points out the weaknesses of the current MEA-based in vitro BCIs and outlines future directions.

This article aims to provide (1) a general overview of the design for high-performance MEAs facilitating brain–computer interaction; (2) a step-by-step guide to analyzing extracellular neural signals; (3) examples of the stimulation paradigm for modulating brain activity in vitro; and (4) case studies illustrating applications of ex vivo bidirectional BCI.

### The need for developing in vitro bidirectional BCIs

**Fig. 2:**
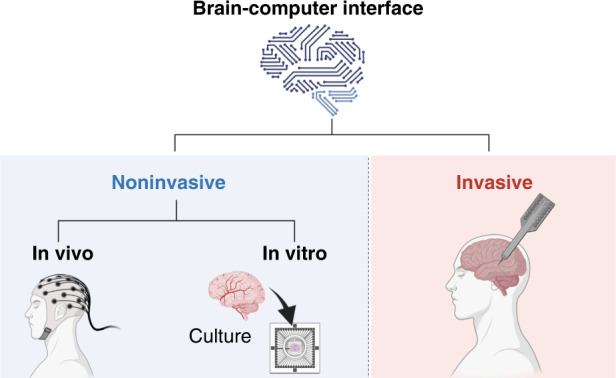
The commonly accepted classifications of BCIs. Existing BCIs are divided into invasive BCIs and noninvasive BCIs based on how the signals are acquired. Invasive BCIs require surgery to implant electronic devices into the brain. A noninvasive BCI can be achieved by fixing the devices outside the skull or directly using brain cells ex vivo with the help of cell culture technology. According to the subject of the research, noninvasive BCIs can be divided into in vivo BCIs and in vitro BCIs (created with http://BioRender.com)

The past decades have witnessed a surge in the applications of BCIs encompassing recovering and enhancing brain functions. Although the current use of BCIs is limited to patients, the ultimate goal is to also have healthy people take advantage of the benefits of BCIs. Sorting vast types of BCIs into different categories is necessary (the commonly accepted classification is shown in Fig. 2). For example, emphasizing the direction of signals (brain to computer, computer to brain, or bidirectional), the placements of the devices (invasive, noninvasive), or the source of neuronal signals (electroencephalogram (EEG), electrocorticogram (ECoG), intracranial EEG (also known as micro EEG or depth EEG; LFP/spike)). In particular, noninvasive BCIs can be divided into in vivo and in vitro systems according to the research objective.

The main challenge in BCI technology is maximizing the usage of the brain with minimized damage to realize stable brain–computer communication, which is why there has been focus on in vitro bidirectional BCIs. Two of the most prominent advantages of in vitro bidirectional BCIs are the introduction of neuromodulation and the reliability of in vitro systems.

Current BCIs embrace neuromodulation to achieve bidirectional communication, not only focusing on data acquisition as in the past. The two-way interactions between the brain and computer have provided insight into brain-to-brain connections, maximizing the efficiency of information exchange. At the same time, novel bidirectional BCIs integrate real-time feedback to modulate physiological and pathological neural activities, which is urgently needed to assist precise and personalized treatments in neurodegenerative diseases such as Parkinson’s and epilepsy^14^. The widely accepted mode of modulation, electric stimulation (ES), has been explored as a therapeutic avenue in artificially generating sensory input and restoring motor function, already utilized by technologies such as deep brain stimulation, cochlear implants, and visual simulation^17,18^.

In addition, noninvasive in vitro BCIs using cultured brain cells not only eliminates the need to penetrate the brain but also provides authenticity, accuracy and a high signal-to-noise ratio. Important factors are the reproducibility and controllability of the in vitro neural culture^19^. Therefore, simplified in vitro models have become great assets for clinical applications to identify the functions and mechanisms of various electrical stimuli, avoiding vain efforts based on “trial and error” and taking safety and efficacy into consideration^20^.

Combining these advantages, in vitro bidirectional BCIs are worthy of attention, particularly the popular MEA-based BCI. At the forefront of bioelectronics, MEA-related neuroscience studies are committed to introducing novel materials, constructing elaborate structures and integrating new technologies for better interplay between neurons and electronics. And the utilization of nanomaterials improves the overall properties of the MEA and is highlighted here.

### Using MEAs as BCIs due to the attributes of the neural network in vitro

**Fig. 3:**
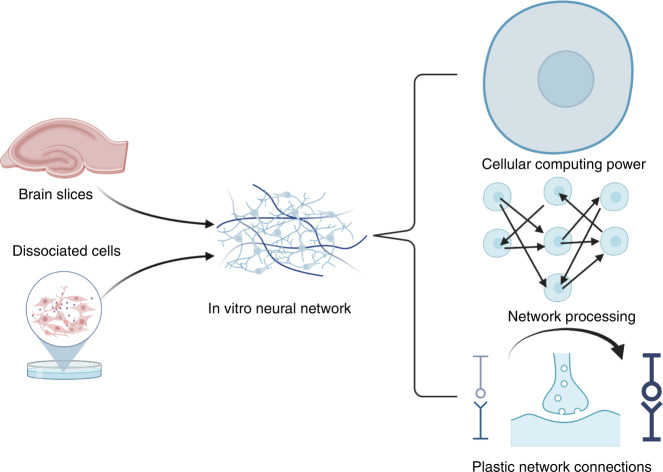
Schematic representation of the characteristics of an in vitro neural network. In vitro neural tissue is primed to handle complex tasks because individual neurons, which store and process information, can perform nonlinear logic operations in parallel. Neurons form networks through synaptic connections with highly conservative network modules (microcircuits/motifs) to realize information sharing, transmission and feedback. The network connections have plasticity, which can strengthen or weaken the connection strength between neurons through variable synapses to adaptively change the network structures and improve the efficiency of neurocomputing (created with http://BioRender.com)

In vitro neuronal culture comes from brain slices or organoids derived from dissociated neurons or stem cells (Fig. 3). Ex vivo systems originating from multiple species conserve the basic properties of in vivo systems, such as cellular computing power, network processing capacity, and plasticity, and they provide additional benefits, such as controllability. To understand and employ a sophisticated computing system, an MEA with high biocompatibility and reliable stability is needed, catering to the characteristics of natural neural networks.

First, the electrophysiological records of in vitro neural culture appear consistent with those in vivo^21,22^, which is why MEAs are widely applied to detect electrical information from ex vivo brain tissues. Using MEAs, the electrical signals generated following routine neuronal activities are easy to read and quantify without fluorescent dyes or transgenes^23^. Different cultures have distinctive properties: acute slices retain sophisticated structures of natural neural networks, while the cell types and the tissue density of slices might cause the tissue section to undergo functional degradation or early death; and dissociated neural cultures generally can be preserved much longer than slices, and the density and complexity of the formed neural network can be easily controlled by the implanted cell types and ratio, culture density, incubation time and cultivation conditions^24^, yet the formation of mature networks takes time. Regardless of the cell or tissue source, a stable and nontoxic MEA interface is required.

Neuronal circuits intrinsically hold outstanding characteristics for encoding temporal and spatial relationships, as well as performing functions in memory and learning^25–27^, due to natural evolutionary optimization^28^. Suitable technologies, such as MEAs, are needed to reveal and utilize the underlying mechanisms. MEAs, which can achieve multichannel parallel recording, are outstanding research tools to investigate individual neurons functioning in isolation and the information processing by multiple neurons interacting with each other through specific synaptic motifs^29^ (recurring nontrivial interconnection patterns), a phenomenon detailed by brain connectomics^28,30^.

In addition, there is tangible evidence that neural networks inherently have the characteristics of small-world networks: almost every neuron is linked with each other through only a few connections^31,32^. So, adding countable functional connections can revolutionize inhomogeneous neuronal networks^33^. Applying ES to a neural network, imitating endogenous electrical signals, can alter the connection structure and thus the network^34^. Therefore, realizing the overall control of the network with ES is feasible, which is why it is common to use MEAs for the nondestructive and reversible neuromodulation via ES^35,36^.

Considering the attributes of isolated neural networks, MEAs are preferable for their bidirectional communication compared with other available technologies. This review mainly focuses on monitoring and modulating neural networks on MEAs. Special attention is given to the analysis of multisite neuronal firing activities because valuable multichannel data are difficult to acquire by other recording methods.

## Advancements in the fabrication of nanomaterial-based MEAs for in vitro bidirectional BCIs

Here, the design principles and fabrication processes of the MEA are introduced, and emerging materials and structures used in fabrication are reviewed specifically. Then, nanomaterial-modified electrodes are classified into four categories; in vitro bidirectional BCIs can be improved by nanomaterial-based modification because it comprehensively enhances the performance of the electrodes. The involvement of nanomaterials in 3D-printed MEAs is briefly introduced.

### Design principle of MEAs

MEAs and the neurons adhered to them constitute the typical biohybrid model for interrogating and modulating neural pathways. The key component is the neural microelectrodes, which are capable of electrophysiological and electrochemical detection. Distinguished from other tissue-level recording methods (e.g., EEG, ECoG, magnetic resonance imaging), MEA records cell-scale charge transfer and actions at the same level, providing neuroscientists with an unprecedented perspective inside the spatiotemporal dynamics of the brain. Often, multisite spikes and LFPs are sampled from an MEA after amplification, filtering and sorting. Spikes or action potentials (APs), recognized as a detected voltage potential above a threshold, are the most typical and fundamental activity of neurons. LFPs originate from synchronized synaptic currents, which are equal to the average signals from clustered neurons^11^.

There are three significant criteria for evaluating MEAs coupled with dissociated neurons: scale, resolution and biocompatibility^37^. That is, MEAs that possess many channels and high spatiotemporal resolution are preferred, especially when the MEAs can perform long-term continuous probing on live organisms with abiotic stability.

### Fabrication of a typical microelectromechanical system (MEMS)-based MEA

**Fig. 4:**
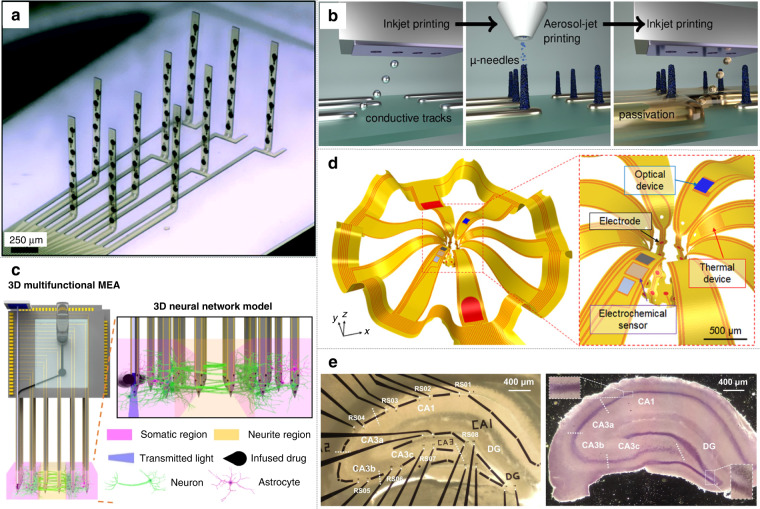
Designs of advanced MEAs. **a** A flexible 3D MEA. Reproduced from ref. ^8^ (2020, CC BY 3.0). **b** Printed μ-needle MEA using conductive polymer ink^69^. Copyright 2019 by American Chemical Society. Reproduced with permission. **c** 3D high-density multifunctional MEA. Reproduced with permission from ref. ^53^ (2021, CC BY 4.0). **d** A soft multifunctional 3D MEA, which is compliant with a neural spheroid. Reproduced with permission from ref. ^54^ (2021, CC BY 4.0). **e** An MEA exclusively made for Hippocampal slices^21^. Copyright 2020 by Elsevier. Reproduced with permission

**Fig. 5:**
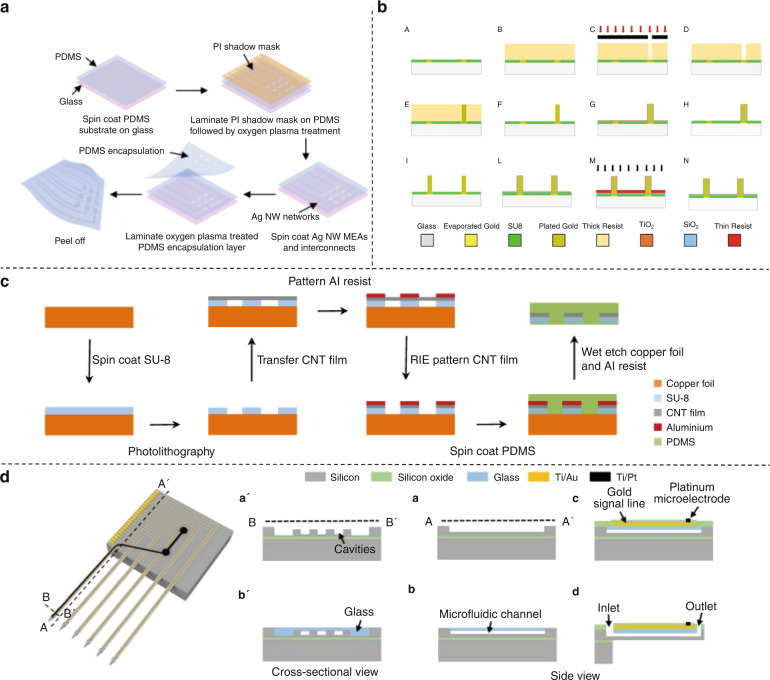
Novel MEA fabrication methods. **a** Fabrication steps for the stretchable and transparent AgNW MEA and interconnects. Reproduced from ref. ^46^ (2021, CC BY 4.0). **b** Fabrication steps for an easy-to-fabricate 3D MEA^50^. Copyright 2020 by IOP Publishing Ltd. Reproduced with permission. **c** Fabrication process of stretchable, transparent CNT MEA^45^. Copyright 2018 by American Chemical Society. Reproduced with permission. **d** Fabrication steps for a multifunctional MEA with optical stimulation and drug delivery. Reproduced from ref. ^53^ (2021, CC BY 4.0)

The traditional structure of MEAs based on MEMS technology comprises (i) a base layer where ubiquitous conductive materials such as gold, titanium, and platinum can be attached and deposited, (ii) a patterned metal layer as electrodes for transduction from bioelectrical signals to machine-processable electrical signals (and the reverse, for stimulation), and (iii) an insulation layer protecting internal interconnection responsible for the size of the electrode. Devices (Fig. 4) and corresponding manufacturing technology (Fig. 5) are constantly updated with lithography as the foundation.

Comprehensive and feasible characterizations of MEA are necessary, ensuring that the devices meet the demands for recording and stimulation^38^. To quantify the performance of the electrodes, typically, the impedance, charge storage capacity (CSC), charge injection capacity (CIC) or charge injection limits (CIL), water reduction potential and oxidation potential (water window) and stability should be determined^39–41^.

In particular, the impedance is the most important: when the densely arranged microelectrodes reach the equivalent size of the cell to achieve large-scale detection, the noise and signal attenuation depend on impedance. Because of the frequency-dependent nature of neural electrophysiological signals, commonly, the impedance magnitude is taken at 1 kHz (center frequency of spike activity) as a representative figure from Bode plots displaying impedance against frequency using electrochemical impedance spectroscopy^16^. High CSC, CIC or CIL are the determinants of effective neuromodulation since the regulation involves the transition from electron flow to ion flow at the electrode–tissue interface via Faradaic reactions and/or the charging and discharging behaviors on the electrochemical double layer around the microelectrodes. The CSC refers to the maximum charge the electrode-electrolyte interface can hold within the water window determined via cyclic voltammetry^16^. This parameter is closely related to the electrochemical surface area, taking the microstructure into consideration. Thus, an increased CSC indicates that the electrochemical reactions on the improved electrode can be more efficient. The CIC or CIL, defined as the maximum injected charge per unit area from the electrodes, is the limit where the delivered waveform polarizes the electrode without irreversible reactions measured by the biphasic current pulsing. For stimulation, the CIC or CIL is crucial to prevent damage to the electrical–biological interface by avoiding irreversible Faradaic reactions. Generally, the higher the CSC is, the higher the CIC or CIL.

### Emerging materials and structures of MEAs for in vitro neural culture

For cellular recording and stimulation, high-performance MEAs must meet a number of requirements at the same time. Basically, good electrical characterization of an MEA is necessary to realize probing with a high signal-to-noise ratio. Since the cells are grown directly on the electrodes and the distance between neurons and the device is related to signal strength, the morphology of the neuron–electrode interface also plays a decisive role. Meanwhile, high biocompatibility and weak biological toxicity are prerequisites for cell adhesion as well as neural signal readout. To obtain superior MEA characteristics, innovations in materials and structures have received special attention.

#### Materials

Various materials have been recently introduced in the base layer, conducting layer and insulation layer. For the base layer, common rigid substrates are still popular, while flexible polyimide-based probes, involving parylene, polyimide, polydimethylsiloxane (PDMS) and hydrogels, are gaining recognition for their ability to noninvasively interrogate a 3D culture of neurons with bidirectional functionality^42^. For conducting layers, nonmetallic materials such as graphene have become intriguing electrode materials due to their excellent electrochemical properties, high flexibility and necessary biocompatibility^43^. Specially treated metal materials also have unexpected gains; for example, holey gold has extraordinary transparency^44^. The transparency makes it possible to observe morphological changes during measurements, and thus the system can facilitate simultaneous optical and electrical regulation. Other transparent materials, such as indium tin oxide (ITO), carbon nanotubes (CNTs)^45^, and silver nanowires (AgNWs)^46^, have attracted considerable attention. ITO is both transparent and highly biocompatible, and the other two materials are ideal candidates for building flexible and stretchable devices. More information about the modification of the conducting layer will be discussed later. However, the insulation layer to protect the conducting wires, which is critical for defining the electrode area and acquiring cell signals, remains the most neglected and awaits innovation^47^.

Surface treatment is the last step before seeding cells. Usually, the MEA is plated with poly-d-lysine or poly ethylene imine (PEI). Then, laminin, found in the extracellular matrix, is placed directly over the electrodes to provide a focal substrate for cell adhesion, thus increasing the quality of the seal^25^. A recent study found that neural assemblies on MEAs with poly-dl-ornithine coating resulted in better survival and improved electric characteristics compared to PEI coating^48^. Additionally, novel hydrogels enhance biocompatibility and thus are used as the interface between electrodes and cells^7^.

#### Structure

There are advancements in the microstructure and macrostructure of bioelectronic interfaces. In microstructure, standard planar electrodes have evolved into raised 3D electrodes, which can promote the engulfment into the cell, achieving concurrent extracellular and intracellular recording. Methods abound, one of which is to bond gold microwires with diameters of 17 μm or 25 µm onto the surface of the planar electrodes^49^. In addition, a facile height-controllable fabrication method for gold pillar-shaped electroplated microstructures was developed^50^. Vertical nanowire MEAs grown mainly by focused ion beam deposition or other common techniques also have high aspect ratios^51^. The most promising 3D vertical electrode is the mushroom-shaped microelectrode, whose unique morphology promotes engulfment by cells without triggering cell repair mechanisms^52^. Traditional planar electrodes only record neural activity from the outside of samples, while 3D electrodes can collect and mediate signals from the inside, offering alternatives to monitor activity with higher fidelity in developed neural tissues such as brain slices. For macrostructure, flexible MEMS technology allows planar MEAs to have customized shapes. Advances in materials sciences facilitate the stacking and bonding of 2D MEAs to build a discrete 3D MEA. An MEA specific to the shape of a hippocampus slice was fabricated (Fig. 4e)^21^. The conformal MEA collected information from precise regions of the slice and successfully gained the spatial dynamics recording of epileptic discharges^21^. The subtle arrangement of electrode sites follows the slice’s structure, acquiring the maximum amount of information with the minimum number of channels. In another study, a 3D MEA had an increased number of channels because it was assembled from three 2D multifunctional MEAs (Fig. 4c)^53^. With the help of flexible materials, these discrete 2D electrodes can be manufactured as a whole (Fig. 4a)^8^. Mechanically actuated flexible 3D polyimide probe arrays can be aligned vertically because of their plastic hinge regions^8^. Additionally, flexible multifunctional 3D framework-like neural interfaces have been fabricated using multilayer mechanically guided assembly, whose geometries perfectly match cortical spheroids (Fig. 4d)^54^.

### Nanomaterial modification of high-performance MEAs

**Fig. 6:**
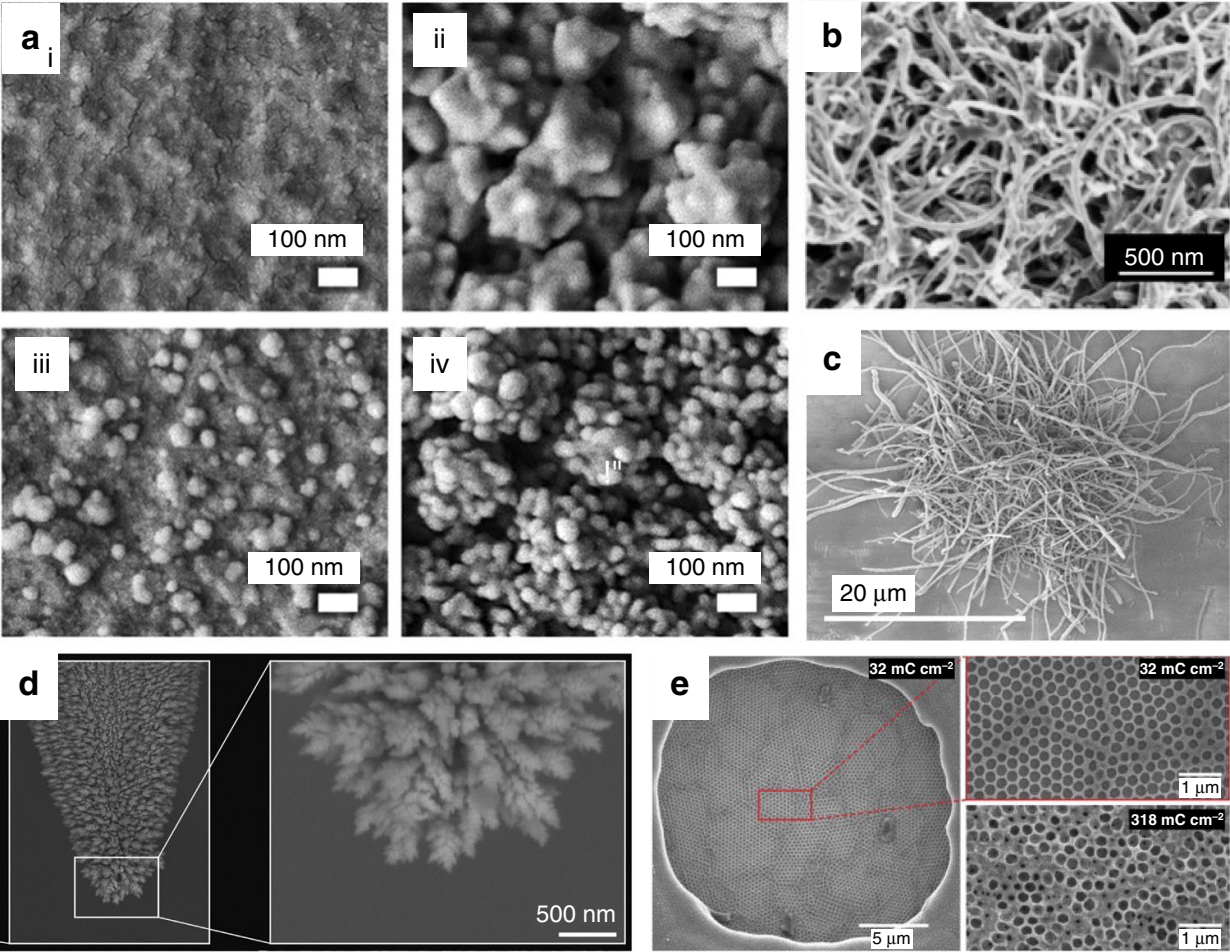
Morphological characterization of nanomaterials to modify MEAs. SEM images of **a (i)** bare Au microelectrode; **(ii)** bumpy Au nanostructure deposited on the microelectrode; **(iii)** Au nanoprotrusions deposited on the microelectrode; **(iv)** hierarchical Au nanostructure deposited on the microelectrode^58^. Copyright 2021 by American Chemical Society. Reproduced with permission. **b** MWCNT/PEDOT:PSS-modified microelectrode^21^. Copyright 2020 by Elsevier. Reproduced with permission. **c** CNT-PEDOT-modified microelectrodes^55^. Copyright 2020 by Elsevier. Reproduced with permission. **d** nanoPt-modified microelectrode^57^. Copyright 2020 by American Chemical Society. Reproduced with permission. **e** Macroporous PEDOT/PSS structures on a microelectrode at 32 mC cm^−2^ and 318 mC cm^−2^ deposition charge densities^56^. Copyright 2019 by Elsevier. Reproduced with permission

Methods for improving the performance of MEAs are abundant, one of which is nanotechnology^14^. Nanomaterials have been widely used in microsystems to enhance specialized electrochemical properties and moderate adverse biological reactions^21,55–58^ (Fig. 6). When the size of the material reaches the nanoscale, it shows a greater atomic utilization, higher charge conduction efficiency and higher metal center activity than larger scale structures, which is suitable for electrochemical sensing with outstanding conductivity, optical properties and strength^14^. For micron-level research objects such as cells, nanoparticles eliminate foreign body responses through interactions with proteins, cells and tissues, and they can even enter the cells to perform their function^3^.

For neural electrodes, nanomaterial coating increases the roughness and porosity of the electrode and thus provides a large electrochemically active surface area with minimal geometrical dimensions and a better charge injection capacity within the limit of the electron-ion transport rate^5^. Flexible and compliant nanoscale materials on electrodes seamlessly integrate with neural tissue and promote intimate neural interactions, therefore mitigating the effects of noise^14^. In bidirectional communication, nanoparticles work as nanoscale converters to transform the applied stimuli into the modality of signals that cells can identify and respond to^57^.

A fairly wide range of modification materials has been developed (Table 1). We divided the popular modifications into four categories: organic polymers, metal materials, inorganic nonmetallic materials and nanocomposite materials. Of course, distinctions exist, but similarities are found in their effects on electrochemical performance and biocompatibility. The types of modifications are also distinguished by their restrictions in long-term applications and repeatability (achieving identical quality of interrogation and modulation across multiple electrodes working in parallel). Interestingly, in regard to electrode modification, it is not enough to consider the material alone; the nanostructure formation and corresponding fabrication methods also have an impact.

**Table 1:** Property comparison of representative nanomaterial modifications for bidirectional neural interfaces in the last 3 years

Material	Fabrication	Diameter (μm)	CIC/CIL (mC/cm^2^)	Cathodic CSC	Impedance	Biological experiment	Reference
cGO/PEDOT:PSS	Electrodeposited	30	3.11 ± 0.25	7.53 ± 0.34 mC/cm^2^	7.26 ± 0.29 kΩ (1 kHz)	In vitro	^40^
Nanostructured platinum	Electrodeposited	35	3.0 ± 0.1	51.3 ± 0.2 nC	66.71 ± 0.44 kΩ (100 Hz)	In vivo	^57^
PEDOT:PSS/ PtNPs	Electrodeposited	30	4.37 ± 0.22	14.84 ± 2.72 mC/cm^2^	10.94 ± 0.49 kΩ; −12.54 ± 0.51° (1k Hz)	In vitro	^39^
PEDOT:CNF	Electrodeposited	20	10.03	7.89 mC/cm^2^	1.28 MΩ/μm^2^ (1k Hz)	In vitro	^55^
PEDOT:PSS	Electrodeposited	40	/	31.5 ± 1.3 mC /cm^2^	9.4 ± 0.9 kΩ (1k Hz)	In vitro and in vivo	^42^
Pt-Ir	Electrodeposited	75	3.85	12.5 + 0.75 mC/cm^2^	50 ± 9 kΩ (1k Hz)	In vivo	^116^

#### Organic polymer nanomaterial modification

Conducting polymer coatings have been widely used to improve neuronal microelectrode performance in bicommunication^59^. Conductive polymers are well matched to biological systems and do not disrupt the native tissue due to their carbon backbones, high biocompatibility and long-term stability^59^. While they do not offer mechanical advantages over inorganic coatings, they do contribute to lower impedance and higher CSC. Improvements are ongoing to avoid delamination, which appears under repeated tests in saline solution.

In organic electronics, poly(3,4-ethylenedioxythiophene):poly(styrene sulfonate) (PEDOT:PSS) is the most popular conducting polymer. Devices fabricated from PEDOT:PSS exhibit outstanding CSC and CIL^55^. It has been shown that microporous PEDOT:PSS CP provides subtle gains in parameters characterizing neuronal recording and stimulation capability^56^. As a better substitute, PEDOT with Nafion dopant possesses higher capacitance and lower cytotoxicity^60,61^. The unsolved problem is that the adhesion of PEDOT:PSS to most widely used substrates is rather low.

It is also worth noting that conducting polymer hydrogels made of PEDOT, PEG, poly(vinyl alcohol), and poly(acrylic acid), among others, have a three-dimensional network structure of polymer chains for retaining water^59^. The soft consistency, high porosity and high water content perfectly mimic living tissues, which makes hydrogels attractive for minimally invasive neural prostheses to reduce mechanical mismatch. The soft coating significantly alleviates the problems of hard devices, such as high impedance and strong foreign body reaction. Moreover, hydrogel coatings can wrap a variety of nanoparticles, forming nanocomposites^59^.

#### Metal nanomaterial modification

Metal nanomaterials with high dielectric constants have always been the first choice for electrode modification. Sputter deposition, laser roughening, and electrodeposition are valid fabrication methods. Compared to conventional metal conductors, the nanometer-sized features of most metals, such as platinum, iridium oxide or titanium nitride, lead to enhanced charge injection limits for neural stimulation. However, the lack of biocompatibility and corrosion resistance prevent the devices from being stably used for monitoring over time.

The optimization of the nano metal materials is achieved through rare materials and novel structures. Precious metals are promising candidates for stimulating electrodes due to their high charge injection limit and corrosion resistance, and liquid metals and shape memory alloys may adapt to the complex and soft environment of organisms. For traditional metal nanoparticles such as platinum (Pt) and gold (Au), novel microstructures rejuvenate them: laser-roughened Pt and microporous Pt have been used to fabricate stable electrodes with low threshold potential and low impedance^57^, and a Au hierarchical nanostructure was deposited to improve the electrochemical surface area in a more effective manner^58^. In particular, quasi-1D metal nanowires with substantial length and ductility facilitate a decrease in resistance and an increase in the complexity of the spatial structure^62^.

#### Inorganic nonmetallic nanomaterial modification

Inorganic nonmetallic materials for neural electrode modification are mainly carbon-based materials, such as carbon nanotubes (CNTs), multiwall carbon nanotubes (MWCNTs), carbon nanofibers (CNFs), graphene, and porous graphene. They offer intriguing properties for neural recording, such as reduced impedance, reliable electrochemical stability, fast electron transfer kinetics and biocompatibility. These materials are also known to feature a wider electrochemical potential window and a high CIC, which is beneficial for cellular stimulation purposes^17^. In particular, there is potential for electrochemical usage. Carbon-based electrodes have been accepted as the gold standard for fast scan cyclic voltammetry to detect dopamine^63^.

Specifically, graphene substrates can enhance the viability of neurons and the length of neurites but with a high error rate and poor uniformity in handling and fabrication^44^. Porous graphene reduces the stacking between graphene layers and gains the advantages of porous materials. CNTs are the most frequently used carbon materials for neural applications, and MWCNTs provide easier preparation, lower cost and increased porosity. CNFs are gaining attention because of their extraordinary strength, large surface area, biosafety and stability. Carbon atoms with different binding modes can form other microstructures, such as large grains in single-layer graphene (LG-SLG). LG-SLG via chemical vapor deposition was put forward and applied successfully to MEAs, where large synchronous neural activities were recorded in an earlier development stage^64^.

#### Nanocomposite modification

Nanocomposites are combinations of nanoparticles. Some associations can yield synergistic effects, while others cannot^39^. For a positive example, CNFs mixed with PEDOT generated a desired synergetic effect, resulting in a microelectrode interface with lower impedance^55^. The good analytical performances of PEDOT:CNF composites come from the effective electronic exchange and the available reactive surface, which help catalyze the electron transfer reaction. The deficiency of the nanocomposites is the performance degradation following delamination, largely due to free-standing nanoscale components. For stability, strong mechanical characteristics and high electrical performance, ‘dead’ volumes or surfaces and the inhomogeneous distributions of phases should be avoided^65^. Nonconducting elastomeric biomaterials and conducting hydrogels are promising candidates to serve as matrices for conducting fillers to produce composite coatings with favorable electrochemical characteristics and biocompatibility^62^.

### Fabrication of novel 3D-printed MEAs using nanomaterials

Unlike conventional manufacturing, 3D printing with nanomaterials exhibits excellent capabilities in fabricating exquisite mini stereostructures without clean rooms in a programmable manner, which further advances the design of MEAs.

Similar to nanomaterial modification, a wide range of materials have been employed. PEDOT:PSS-based conducting polymer ink was used in 3D printing to fabricate a soft neural probe capable of single-unit recording (diameter: 30 µm; impedance: 50–150 kΩ (1 kHz))^66^. Additionally, an MEA utilizing conductive nanoporous carbon ink was manufactured, whose structural resolution was comparable to those fabricated in cleanroom. The nanoporous carbon microelectrodes exhibited high specific capacitance and recorded the spontaneous activity of HL-1 cells extracellularly^67^. Moreover, highly controlled directional printing of quasi-1D metal nanowires may promote the development of 3D MEAs^68^. Composites with superior properties cannot be ignored. The function and biocompatibility of 3D needle-like electrodes (diameter: 10 ± 2 μm; impedance: 128 ± 22 kΩ (1 kHz); height: 33 ± 4 μm) using nanocomposite ink combining PEDOT:PSS and MWCNTs were verified through proof-of-principle biological experiments (Fig. 4b)^69^.

### The future of nanomaterial-based MEAs for in vitro bidirectional BCIs

The potential design, fabrication and characterization of nanomaterial-based MEAs for in vitro bidirectional BCIs are presented here. The renewal of MEAs depends on the material and structure, and a more affordable and more useful device is a consistent pursuit. There is a higher desire for transformable MEAs that can adapt to the morphological changes that come with the growth and development of brain tissue in vitro during long-term culture^70^. The joint application of multiple technologies to the design of MEA is also attractive. Multifunctional electrodes combined with optogenetic technology, microfluidic technology, flexible electronics and other emerging technologies can meet the high-level needs of comprehensive experiments involving multiple conditions and factors.

Nanomaterial-modified MEAs using MEMS technology have widely served as in vitro bidirectional BCIs to collect neural activities and realize dynamic feedback control. Nanomaterial-based 3D printing of MEAs is a promising method. Other fabrication methods, such as complementary metal-oxide-semiconductor (CMOS) technology^71^ and thin film transistor (TFT) technology^72^, are hopefully to be improved by nanomaterials.

Noticeably, the best tests for comparing electrodes are usually in 3-electrode cells with phosphate buffered or physiological saline. However, the electrolyte solution is too simple to compare to a multicomponent mixture in a natural biological environment. Therefore, the values measured are merely approximations of the microelectrode behavior wrapped in a neural network. Moreover, protocols indicating the variation in these parameters over time after implantation remain to be explored.

## Bidirectional communications with neural networks on MEAs

### Introduction of electrical signal exchange between an MEA and neurons in vitro

Classical experiments involving communication with neuronal cells date from the early 1790s, when Luigi Galvani first stimulated frog legs^73^. However, to date, the interactions between the input electric pulse sequence and collected electric signals have been difficult to interpret.

ES depolarizes the membranes in close proximity as a controllable and reversible switching, which is clearly beneficial to work in the interactions with neurons. Simultaneous multisite recording of cellular neural signals, which is difficult to obtain without an MEA, is an important prerequisite for interpretation of neuronal behaviors, which is a more complex task than judging whether animals or humans behave as expected. Thus, the data processing of electrical activity captured by MEAs and the corresponding stimulation paradigms deserve careful study.

### Decoding: analysis of neural signals from the cellular level to the network level

Decoding neural signals is fascinating but complicated. Ex vivo brain tissue from various species has the capability to process multichannel data and exhibit the universal fundamental property of plasticity. Inside in vitro neural networks, not only can individual neurons run as sophisticated information-processing units, but their connection patterns through synapses also enable the execution of computational tasks^74^. Remarkably, the network dynamic patterns represent a specific operation mode, revealing memory traces, information flow and so on^75^. This section provides a step-by-step guide for those who want to decode the characteristic activity of in vitro neural cultures, including the analysis of the following: cellular activity, population activity, network dynamics and other phenomena.

#### Cellular activity

The basic electrophysiological features describing neuronal activity extracted from MEA recordings are spikes and bursts^76,77^. A spike is the action potential (AP) detected by the MEA when neurons fire, and a group of temporally overlapping spikes (a series of APs) firing at a frequency much higher than the mean firing rate (MFR) is termed a burst. Both activities are intrinsic properties of neural networks^78,79^.

Analysis usually starts with the basic quantitative parameters of spikes. Normally, MFR is paramount due to the intrinsic variability of neural activities. The interspike interval is used to characterize the firing pattern and discern regular and irregular firing. For mature neurons, spontaneous neuronal firing occurs in a periodic fashion, patterning with intervals^80^. Periodicity, the repetition of spike patterns, can be evaluated using autocorrelation or Welch’s periodogram. A poststimulus time histogram of spikes can also be computed to investigate the effects of stimuli on neurons^81^. More electrophysiological features can be taken into consideration^78^. Burst analysis is similar.

Further analysis includes the classification of signals, unveiling the inner heterogeneity for comprehensive understanding. For example, spikes from putative inhibitory and excitatory neurons are routinely classified based on the half-peak waveform width of the largest waveform and the polarity for each unit^82^ or the Fano factor (the ratio between spike-count variance and mean)^80^. Recently, multifactor cluster analysis was introduced in the classification of spikes^83^, and unsupervised machine learning methods have been applied to cluster spike patterns^84^ and burst patterns^85^.

#### Population activity

Brain functions come from the cooperative activities of multiple neural clusters, which is why population activity is important. Additionally, decoding large-scale neuronal dynamics provides robustness and reliability for revealing normal physiological activities and abnormal pathological phenomena^86^. The average electrical activities of neurons around the electrodes are represented as LFPs or the overall spike characteristics. LFP is the dominant signal acquired by diversified electrophysiological recording techniques, accompanied by many mathematical analysis methods^87^. Therefore, attention is given to collective activities represented by multichannel spike trains, which are difficult to record without an MEA.

To characterize the complex behavior of population-wide activities, spontaneous events and synchronization must be taken into consideration. When the majority of the neurons in the biological network fire simultaneously, the dramatic reverberating neural event is called a ‘network burst’, ‘spontaneous burst’, ‘super burst’ or ‘population burst’, abbreviated ‘SB’ in this article. The bursts of elevated population activity, correlated in space and time, are supposed to be tied to neuronal avalanches and synchronized oscillation^88^. The hallmarks of neuronal spontaneous activity are intermittent SBs, separated by periods of silent activity, and the intervals between SBs are distributed approximately lognormally^89^. To assess changes in network bursts, the following parameters were analyzed: SB duration, interSB interval and SB percentage^80^. Interestingly, some neurons always fire ahead of the SB, as if they trigger the network activity, which are called ‘burst leaders’. SBs are believed to play an essential role in many neural processes, such as generating multifunctional patterns, learning, connecting sensory circuits, and encoding and maintaining memory, and are associated with various neurodegenerative diseases, including epilepsy, Parkinson’s disease, Alzheimer’s disease and schizophrenia^74^.

Different from instantaneous synchronization, synchronous firing refers to when two or more neurons fire in a similar pattern, i.e., sequential synchronization^90^. The cross-correlation between neurons is used to measure the degree of synchronization^80^. The Pearson correlation coefficient is a widespread canonical measure used to verify spike train synchrony. A novel measure of correlation, the spike time tiling coefficient, is also valuable and independent of the object-specific firing rate. Notably, bursts, well-organized neuronal events, appear when large-scale neurons become synchronic^80^. These activities, similar to those observed in vivo, are related to the plasticity and dynamics of the neural network.

#### Network dynamics

**Fig. 7:**
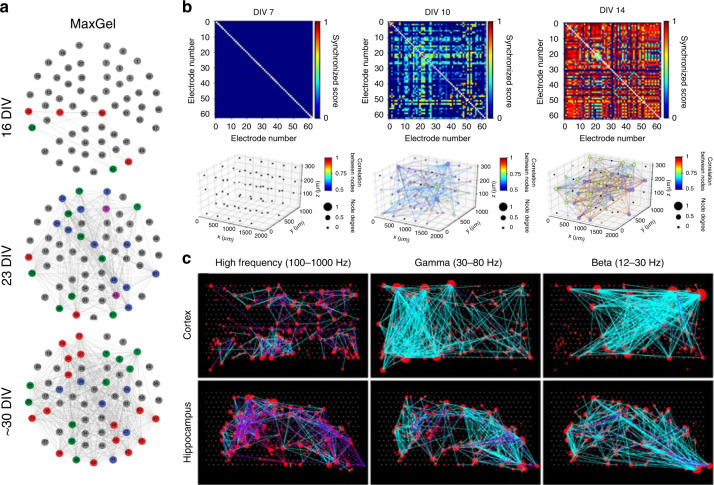
Analysis of neuronal network dynamics. **a** Synchrony and community structure of neural networks formed in the presence of a nontissue-specific brain extracellular matrix (MaxGel) coating. Reproduced with permission from ref. ^115^ (2019, CC BY 4.0). **b** The 3D functional network maps of mature cortical neural networks in vitro over time based on synchronized scores between electrodes. Reproduced with permission from ref. ^53^ (2021, CC BY 4.0). **c** Connectivity map of neural networks of 6 different cortical and hippocampal tissues at three different frequency ranges. Reproduced with permission from ref. ^93^ (2014, CC BY 4.0)

Neurons cultured in vitro can no longer reproduce after differentiation, while the synapses that connect them together are constantly changing. It is the connection that fully mobilizes all cells, allowing individuals to function better. Functional connectivity, distinguished from anatomical connectivity, represents the relationship between dynamic neuronal activities^80^. The measure of modularity, representing the community structure employed in assemblies of functionally distinct clusters, is usually identified in graph-theoretic methods^91^. Neurons or active electrodes can be regarded as a subset of nodes, and the links correspond to the relation between the nodes (Fig. 7).

Researchers focus on the functional connections of the reticular neuronal circuits because they provide a superior proxy of the neuronal reticular topological structure on an MEA^92^. However, the structural network of the grown culture is so redundant that it can hardly benefit research. Unquestionably, finding the correlation between the physical network and defined functional network is difficult but meaningful.

To reveal functional associations, conditional firing probabilities and cross-correlation coefficients provide usable descriptions of relationships between firing activities^64,93,94^. Information theory also helps. Shannon entropy has been used to quantify the effect of the stimuli location variable on the neural response^71^. Mutual information quantifies how much information in one signal is contained in the next, which thus determines the information transmission^88^. To examine information flow, the transfer entropy measure identifies the direction and flux of information transfer^25^. Moreover, all bursts originating from the same electrode, as a leader electrode, are grouped together, and for each follower electrode, the minimum propagation delay (i.e., delay of the first detected spike) is calculated to exhibit network structure^95^. Similarly, compared with a relatively sparsely connected single neural network, when one neural network is interlaced with another, the synaptic latency representing the time required for signal transmission is direct evidence of connectivity^53^. To facilitate the study of network dynamics, handy software toolboxes have been developed to verify the functional network^96,97^.

After building functional networks, the dynamic changes of the network need to be determined to investigate whether functional connections appear, disappear or reappear. Comparing a quantified parameter characterizing population activity is feasible^80^. Based on graph theory, these electrophysiological activities can be mapped to an appropriate coordinate system as points, and the change across periods can be quantified using, for example, the Euclidean distance or Mahalanobis distance^98^. The matrix constructed by the relationship of multichannel data before, during, and after a disturbance can be compared using the similarity index^99^.

#### More analysis

Some factors are not independent from each other; for instance, a phase shift appears between the spike and LFP^100^. More attention should be given to the interconnections between events of multilayers. Machine learning algorithms are adopted in the statistical analysis of neural networks in vitro to integrate cellular electrophysiological features, synchronized population events, network connectivity and others to create a high-fidelity multidimensional profile^78^, which is a valuable guideline.

### Encoding: paradigms of electric stimulation for neuromodulation strategies

The controllable and reversible ES has direct reciprocal connections to the in vitro cultured system and is involved in multiple vital movements^101^. Therefore, ES with different settings applied to various types of cells may have diverse effects. Here, representative paradigms for ES and their functions are listed with key parameters (Table 2). Special attention should be given to stimulus paradigms designed based on Hebbian theory^39^ and the free energy principle^102^, which evoke advanced functions such as learning and memory and make full use of exquisitely connected natural neural networks without affecting internal development.

**Table 2:** Overview of the electric stimulation paradigm and function

Subject	Sites	Frequency	Amplitude	Duration	Function	Ref.
Cortical culture	2	1/30 Hz ~1/2 Hz	60 μA	900 s	To study selective adaptation	^117^
Cortical culture	1, 2	1/50 Hz	0.5–0.7 V	5000 s	To study stimulus-response relations	^89^
HIP culture	7	1/3 Hz	1 V	450 s	To evoke neuronal activity	^80^
HIP culture	2	6 Hz	1 V	3600 s	To evoke interval learning	^25^
HIP slice	1	130 Hz	75% of Max_pp_	<15 s	To inhibit low-Mg-induced epilepsy	^118^
Cortical slice	2	100 Hz	75% of Max_pp_	<5 s	To study induced neural enhancement	^119^
Cortical culture	1	1 Hz	/	20 s	To evoke neuronal activity	^120^
Cortical culture	1	/	750 mV	/	To study the impact of stimulation temporal distribution	^121^
HIP culture	8	0.2 Hz	750 mV	300 s	To study the state-dependence of evoked activity	^71^
HIP culture	1	1 Hz	300 mV	3300 s	To activate learning and memory functions	^39^
HIP culture	2	/	400–800 mV	1200 s	To study selectivity for the injected information	^95^
Cortical culture	2	0.2 Hz	12, 24 and 36 µA	>10 h	To study memory trace formation and consolidation	^75^
SiHa and HeLa culture	1	10 Hz	25 V	/	To enhance transmembrane transport	^122^

Stimulation duration refers to the total time from the first stimulation to the last stimulation

Max_pp_ refers to the maximum intensity of postsynaptic potential

HIP refers to hippocampal

**Fig. 8:**
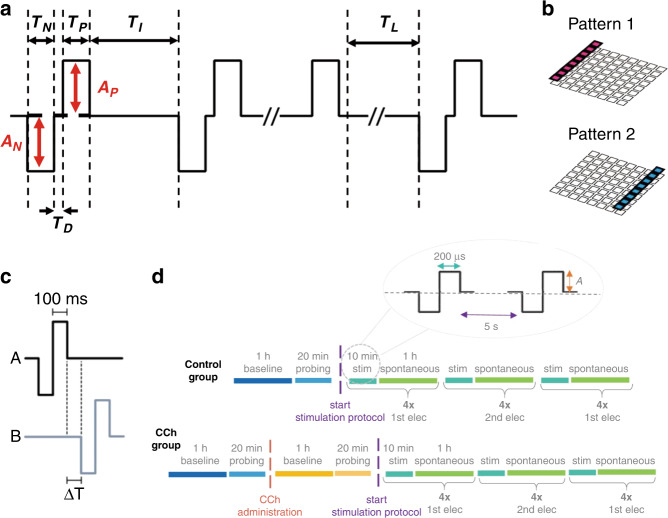
Electric stimulations for neuromodulation. **a** General template with necessary parameters (negative potential duration (*T*_N_), negative potential amplitude (*A*_N_), positive potential duration (*T*_P_), positive potential amplitude (*A*_P_), interval time between two pulses (*T*_I_), delay time (*T*_D_)) describing ES composed of 3 successive electric stimulus sequences, broken down by rest without stimulation. T_L_ represents the time between two stimulus sequences. **b** Two electrode arrangements used in stimulation protocols to evoke neuronal activity. Reproduced with permission from ref. ^80^ (2021, CC BY 4.0). **c** Stimulation pattern combining A and B to train interval delay (Δ*T*). Reproduced with permission from ref. ^25^ (2019, CC BY 4.0). **d** Design of the electric stimulation paradigm for the control group (top) and carbachol (CCh) group (bottom) to form and consolidate memory traces. Reproduced from ref. ^75^ (2021, CC BY 4.0)

Developing new paradigms is conducive to understanding the information processes in the brain^103^. For neuromodulation, cellular activity depends largely on the amplitude and frequency of ES, while other determinable parameters also matter (Fig. 8), such as pulse width^104^, wave form^25^, arrangement of stimulation sites^80^, and stimulation duration^75^. To ensure a reliable stimulation effect, suitable stimulation paradigms following basic design criteria^101^ are pivotal.

### Challenge of bidirectional communication through in vitro BCIs

For quantifying neural activity using an MEA, the combined use of electrochemical signals and electrophysiological signals can provide a more comprehensive perspective. A nanocomposite-modified multifunctional MEA that can perform double-mode sensing successfully detected dopamine exocytosis in human embryonic stem cell-derived dopaminergic neurons (hESC-DDNs), and the differentiation effect was assessed^105^. The leading work greatly promotes the application of hESC-DDNs in clinical practice. In fact, versatile neural interfaces with capabilities including recording, stimulation and electrochemical determination of neurotransmitters depend largely on novel nanostructured materials^55^. In addition, the sensitive recording and instant decoding of neuronal activities are constantly being improved to aid in evaluating the responses to customized stimulation pulses as necessary feedback to benefit bidirectional communication.

Concerning the information input, we ignore indirect electrical coupling, such as implementing an electromagnetic field although parallel plates, field-effect transistors, induction coils or solenoids, generating ultrasound acting on ion channels and using optogenetic tools to manipulate neurons^35,106^. Instead, we focus on direct electrical coupling, which uses electrodes to inject charge through neural electrical interfaces. As indicated earlier, a lot has been achieved through ES. Nevertheless, developing new paradigms for modulation is meaningful, especially those that can be adaptively personalized based on the fluctuations in the characteristics of isolated neural networks^107^. Other breakthroughs lie in increasing the precision of stimulation by limiting the changing electrical field to a certain area^108^.

## Application of in vitro BCIs

**Fig. 9:**
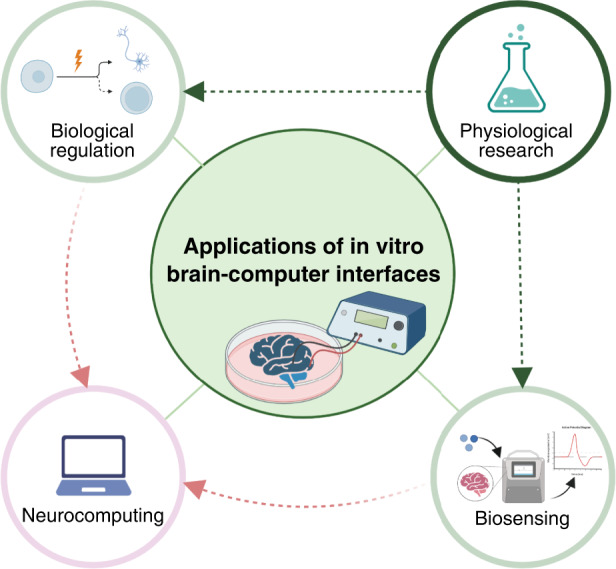
Applications of in vitro BCIs. The fundamental application of in vitro BCIs is performing physiological research on brains-on-chips to clarify basic mechanisms under brain function. The clarified mechanisms can be utilized in biological regulation and biosensing. In vitro bidirectional BCIs combining these two functions provide the foundation for biological neurocomputing, which exploits the plasticity of the isolated brain with high efficiency and low power consumption (created with http://BioRender.com)

The development of in vitro BCIs brings infinite possibilities in four fields in particular: (a) physiological research, (b) biosensing, (c) biological regulation and (d) neurocomputing (Fig. 9). Basically, neural networks on MEAs have the ability to emulate brain patho/physiology like all organs-on-chips, revealing internal mechanisms^109^. On MEAs, the in vitro neuronal network model converts complex or vague mechanical and chemical factors into readable electrical signals through interactions with the microenvironment, which is advantageous. Therefore, MEAs coupled with in vitro brain samples have routinely served as biosensors to evaluate the effects of, for example, chemical drugs^81,110^, biomaterials, and physical forces on brain cells^111^. Moreover, the information extracted through MEAs facilitates biological regulation, such as controlling the differentiation of NSCs^80^ and suppressing epileptiform activity in rat hippocampal slices^112^.

Neurocomputation is the most striking usage because of the exquisite structure and dynamic adaptability of natural neural networks based on synaptic plasticity^113^. A neuronal network is similar to an “organic computer” with intelligence. A series of ESs can encode customized information, and the computation results can be decoded from the detected electric signals. Bidirectional in vitro BCIs have already exerted excellent control capability in robot movements^114^ and demonstrated superior learning ability in playing games^107^. Hopefully, quick and effective encoding and decoding of neural activity can facilitate the connection of several brains from different sources in vitro to manage parallel operation with higher precision.

## Conclusions and future perspective

MEAs are designed with affordability and flexibility and they can be customized tools to modulate neural networks. In particular, the nanoscale roughness and porosity of nanomaterial modification improve cell adhesion, electrochemical reaction area and charge storage capacity, and tissue sealing between nanoparticles and cells yields a high signal-to-noise ratio. In summary, nanomaterial-based MEAs have outstanding strengths for bidirectional BCIs in vitro: (1) they possess inherent and instant bidirectional communication ability through electric signals with millisecond temporal resolution, as well as a gradually growing encoding dictionary and an increasingly standardized decoding path; (2) they acquire information directly from neuronal activity, not indications; (3) they provide a unique global perspective from individual cell to neuronal network; and (4) they offer continuously optimized devices due to the rapid development of related disciplines.

In conclusion, this is a distinctive review centering on in vitro bidirectional BCIs with a broad overview of nanomaterial-based MEAs and neural signal–electric signal translation on MEAs. Interdisciplinary information is collected through bidirectional in vitro BCIs for the application of MEAs affiliated with living organisms in more situations. The unlimited possibilities of the devices are only partly displayed, aiming to inspire interest in the promising field.

**Fig. 10:**
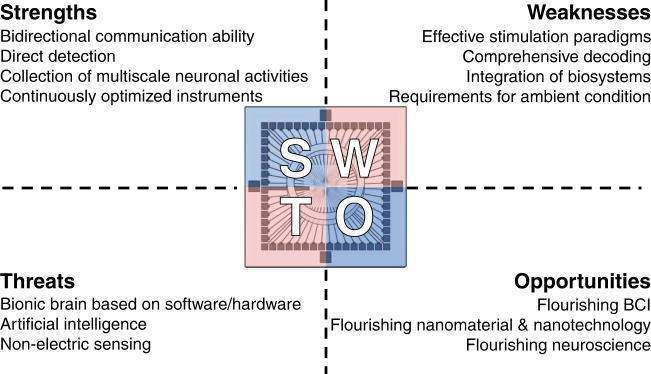
Perspectives about nanomaterial-based MEAs for in vitro bidirectional BCIs. The future development of the field relies on the strengths of the devices, and overcoming the weaknesses of current studies is the main development direction. In addition, the developments of related fields largely influence the future of in vitro bidirectional BCIs as threats and opportunities

To advance the development of bidirectional in vitro BCIs, the problems mainly exist in exploring new and effective stimulation paradigms mimicking sensory input and decoding neural activities in a more comprehensive manner, such as combining electrophysiological signals and electrochemical signals with high-performance BCI (Fig. 10). Nevertheless, better interfaces are needed to achieve seamless integration of biosystems and electronic systems, and the introduction of nanomaterials is an exemplary solution, as suggested in this paper. Moreover, it is necessary to reduce the influence of ambient conditions to cater to unknown and complicated operating conditions, which leads to higher demands for integrated systems maintaining the internal balance of living neural networks. For future work, the focus is employing in vitro brain samples as intriguing central processing units to handle complex tasks in various application scenarios (Fig. 9). Functionally mapping reticular neural circuits in vitro and totally achieving natural human intelligence on chips are the ultimate goal for further application, which requires the cooperation of devices and appropriate analysis methods. The direction for the next-generation of BCIs is pointed out. In particular, the BCIs mentioned in this paper are expected to facilitate direct brain‒brain connection, that is, an open connection between brains, within the same species or even across species.
